# Modeling transcriptional activation changes to Gal4 variants via structure-based computational mutagenesis

**DOI:** 10.7717/peerj.4844

**Published:** 2018-05-29

**Authors:** Majid Masso, Nitin Rao, Purnima Pyarasani

**Affiliations:** Laboratory for Structural Bioinformatics, School of Systems Biology, George Mason University, Manassas, VA, United States of America

**Keywords:** Knowledge-based potential, Variant function prediction, Computational mutagenesis, Structure–function relationships, Machine learning, Gal4

## Abstract

As a DNA binding transcriptional activator, Gal4 promotes the expression of genes responsible for galactose metabolism. The Gal4 protein from *Saccharomyces cerevisiae* (baker’s yeast) has become a model for studying eukaryotic transcriptional activation in general because its regulatory properties mirror those of several eukaryotic organisms, including mammals. Given the availability of a crystallographic structure for Gal4, here we implement an *in silico* mutagenesis technique that makes use of a four-body knowledge-based energy function, in order to empirically quantify the structural impacts associated with single residue substitutions on the Gal4 protein. These results were used to examine the structure-function relationship in Gal4 based on a recently published experimental mutagenesis study, whereby functional changes to a uniformly distributed set of 1,068 single residue Gal4 variants were obtained by measuring their transcriptional activation levels relative to wild-type. A significant correlation was observed between computed (scalar) structural effect data and measured activity values for this collection of single residue Gal4 variants. Additionally, attribute vectors quantifying position-specific environmental impacts were generated for each of the Gal4 variants via computational mutagenesis, and we implemented supervised classification and regression statistical machine learning algorithms to train predictive models of variant Gal4 activity based on these structural changes. All models performed well under cross-validation testing, with balanced accuracy reaching 91% among the classification models, and with the actual and predicted activity values displaying a correlation as high as *r* = 0.80 for the regression models. Reliable predictions of transcriptional activation levels for Gal4 variants that have yet to be studied can be instantly generated by submitting their respective structure-based feature vectors to the trained models for testing. Such a computational pre-screening of Gal4 variants may potentially reduce costs associated with running large-scale mutagenesis experiments.

## Introduction

**Figure 1:**
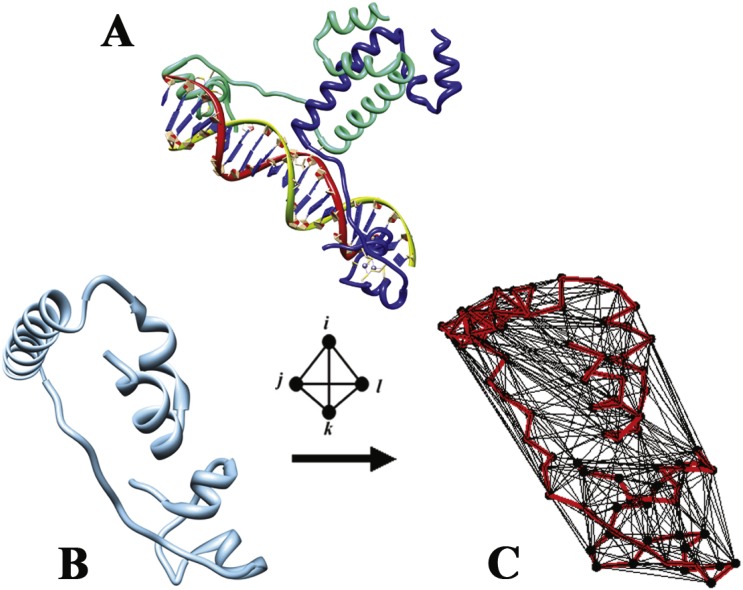
Delaunay tessellation of the Gal4 protein structure. (A) Ribbon diagram of the Gal4 homodimer bound to DNA based on the PDB accession file 3coq, and (B) an isolated ribbon diagram of one Gal4 monomer (chain A) from the same structure. (C) Delaunay tessellation of monomeric Gal4 coarse-grained at the amino acid level. The residues are represented as points by using the coordinates of their C-alpha atoms, and these points serve as the vertices for the tetrahedra generated by the tessellation. The tetrahedral edges are drawn in black, and the triangular faces of the tetrahedra are transparent to enable visualization. A C-alpha trace of the protein chain is outlined in red.

Galactose utilization by *Saccharomyces cerevisiae* (baker’s yeast) requires the orchestrated collaboration of *GAL* gene products for its transport into the cell and subsequent metabolism via glycolysis (Johnston, 1987). The Gal regulon consists of structural (*GAL1*, *GAL2*, *GAL7*, and *GAL10*) and regulatory (*GAL3*, *GAL4*, and *GAL80*) genes, with the GAL4 protein serving as a transcriptional activator for the structural genes which binds upstream activating sequences (UAS_GAL_) located in their promoters (Lohr, Venkov & Zlatanova, 1995; Traven, Jelicic & Sopta, 2006). Functional Gal4 binds DNA as a homodimer, each protein chain containing 881 amino acids with an N-terminal Zn_2_-Cys_6_ binuclear cluster DNA-binding domain (residues 8–40), an extended loop linker (residues 41–49), a dimerization domain (residues 50–96), and two acidic C-terminal activation domains (residues 148–196 and 768–881) (Hong et al., 2008). A complete X-ray crystallographic structure of the Gal4 dimer (residues 8–96) bound to DNA (Fig. 1) was determined at a resolution of 2.4 Å (Hong et al., 2008), and atomic coordinates were deposited in the Protein Data Bank (PDB) under accession code 3coq (Berman et al., 2000). With growth on glucose and in the absence of galactose, Gal4 is inactivated by dimers of the repressor Gal80 protein, which bind the dimeric Gal4 activation domains (Egriboz et al., 2013). The availability of galactose converts the Gal3 protein to a transducer form which competitively binds Gal80 (Egriboz et al., 2013; Lavy et al., 2012), leading to a significant rise in Gal3–Gal80 interactions along with a concomitant decline in Gal80 self-associations, as well as a rapid induction of transcriptional activation by Gal4 via recruitment of coactivators and transcription machinery to promoter regions through its activation domain upon Gal80 dissociation (Egriboz et al., 2013). Extensive studies have revealed this mechanism of transcriptional activation by Gal4 to be conserved among eukaryotes; in particular, Gal4 was shown to activate transcription when expressed in mammalian cells (Traven, Jelicic & Sopta, 2006).

Owing to the modular nature of the DNA-binding and activation domains of Gal4, a recent study used the protein to demonstrate a proof-of-concept for an experimental technique named programmed allelic series (PALS), a site-directed mutagenesis approach using microarray-programmed oligonucleotides (Kitzman et al., 2015). A PALS library was constructed for the Gal4 DNA-binding domain (residues 2–65) which targeted each codon for replacement. The library (fused with a transcriptional activation domain) was then introduced into a two-hybrid reporter strain in which *Gal4* was deleted and the *HIS3* gene was under the control of the *Gal1* promoter; hence growth on a medium lacking histidine was dependent upon the ability of the introduced Gal4 mutant to bind to and activate *HIS3* expression.

Selection for Gal4 function was reported for increasing time points and stringency conditions as a log_2_(effect size) for each single residue Gal4 variant. The aim of this study is to model the functional effects of Gal4 variants under normal biological conditions, which implies moderate growth time and minimally required selection. Hence, the functional data used for analysis in the present work and obtained from the supplementary material in that study correspond to growth over 40 h on synthetic complete (SC) media lacking urea and histidine (data set named SEL_A_40 h, media type SC –ura –his) (Kitzman et al., 2015). The data suggest that Gal4 variants can be categorized by their log_2_(effect size) values as having activity levels that are superior to wild-type (>0.0), equivalent to wild-type ([ − 2.0, 0.0]), or inferior to wild-type (< − 2.0). Since the Gal4 structure with PDB accession code 3coq is missing residues 1–7 (Hong et al., 2008), we were unable to utilize the available Gal4 functional data for single residue substitutions at positions 2–7. Additionally, functional data were not determined for a few Gal4 variants at scattered positions with the PALS approach, leaving a total of 1,084 Gal4 variants with functional data for analysis in this study.

Computational methods for determining the functional consequences to proteins upon single residue replacements incorporate sequence, structure, and/or evolutionary information (Bromberg & Rost, 2007; Sim et al., 2012). The decision as to which protein features to include depends on both the methodology being developed as well as the particular type of functional effect under consideration (e.g., changes to stability, activity, association with a disease state, resistance to a molecular inhibitor, etc.). Furthermore, a model developed for predicting and understanding one type of functional effect generally cannot be used for inferring other consequences to the protein upon mutation. With respect to protein structure, the analysis of known structures in the PDB has led to the development of knowledge-based statistical potentials, many of which are based on pairwise distances, and they have been used successfully for protein structure prediction and assessment (Hamelryck et al., 2010). Only one recently published study to date has reported an *in silico* method to study the functional consequences to Gal4 variants (Pires et al., 2016). Their computational mutagenesis approach used a structure-based statistical potential generated from knowledge of tolerated residue replacements within families of homologous proteins of known structure (Topham, Srinivasan & Blundell, 1997), and the features they generated for the Gal4 variants were combined with machine learning models. This model was previously developed for studying stability changes, and here it led to marginal Gal4 variant predictions; however, predictions improved once this model was combined with four additional models (each also poorly performing on Gal4 variants individually) designed to capture the effects on diverse protein characteristics upon mutation, including changes to protein-protein and protein-nucleic acid interactions. We previously developed a computational technique for quantifying structural changes to proteins upon single residue mutations using a four-body statistical potential, and by combining these data with machine learning algorithms, we developed models for accurately predicting protein stability and activity changes upon mutation (Masso & Vaisman II, 2008; Masso & Vaisman II, 2007). Our method complements that of Pires et al. while relying on a singular approach for generating structure-based Gal4 variant feature vector data for training predictive machine learning models, without the need to combine multiple methods each designed for predicting the effects on a particular trait.

Here we perform a systematic analysis of the structure-function relationship in Gal4 by comparing the experimentally quantified functional effects of single residue Gal4 variants to computationally determined structural impacts due to the mutations. To implement our *in silico* mutagenesis technique, a residue-based coarse-grained representation of Gal4 was generated using the C-alpha coordinates from the native Gal4 X-ray crystallographic structure. The three-dimensional (3D) space occupied by this set of C-alpha points was packed with hundreds of space-filling, non-overlapping tetrahedral tiles using Delaunay tessellation (Fig. 1), a well-established computational geometry technique (De Berg et al., 2008). Every tetrahedron objectively identifies at its four vertices a quadruplet of nearest neighbor Gal4 residues in the structure, and given the tetrahedral packing arrangement in a tessellation, each C-alpha point is generally shared as a vertex among several tetrahedra. A score was assigned to each tetrahedron, based on the quadruplet of residues represented by its C-alpha vertices, by using a previously developed four-body knowledge-based energy function (Masso & Vaisman II, 2008; Masso & Vaisman II, 2014) that consists of tabulated interaction energy scores for all types of residue quadruplets. As detailed in the Methods, these tetrahedral scores are used to calculate both the Gal4 potential energy and an 89-dimensional (89D) profile vector of residue environment scores for all positions in the Gal4 structure with PDB accession code 3coqA. Similar calculations may be obtained for any Gal4 variant by first altering the residue type associated with the corresponding vertex in the structure tessellation. The difference between the mutant and native Gal4 energies, referred to as the *residual score* in this work, empirically quantifies the global change to sequence-structure compatibility upon mutation. Additionally, the difference between the mutant and native Gal4 profile vectors of local residue environment scores is referred to here as a *residual profile*, whose components quantify the relative structural impacts locally at each of the 89 residue positions in Gal4. The Gal4 variant residual score data revealed a significant structure-function correlation, and components of the residual profiles for the variants were used to train machine learning classification and regression models capable of accurately predicting Gal4 variant functional effects.

We previously achieved comparable results with activity data for 372 single residue substitutions of the enzyme thymidylate synthase from *Escherichia coli* (Masso, 2015), which is similarly functional as a homodimer. In that study, the structural data were generated by tessellating a single chain of the protein, and the same approach is initially implemented in the work here with Gal4 (Fig. 1). However, as we report in this work, a reexamination that employs tessellation of the complete homodimeric structure of Gal4 takes into account interactions between residues from both chains and yields additional improvements, particularly with respect to predictions for mutations at dimer interface residue positions. With both thymidylate synthase and Gal4, the variants were each represented using a 27D vector of attributes that included only seven components from their residual profiles, those corresponding to the mutated position and its six nearest neighbors. The remaining 20 features for each variant highlighted additional structure (and sequence) characteristics unique to the local 3D environment of each mutated position. These 27 components, which are outlined below in the Methods, are universal because they describe an identical set of characteristics for all single residue variants regardless of the protein. Variant residual profiles, on the other hand, are protein-specific with regard to both number of components (i.e., length of the sequence in the 3D structure) and characteristics represented by those attributes (i.e., environmental impacts or perturbations at all positions in a particular protein upon mutation). For comparison, we conclude by using the 89D residual profiles of the Gal4 variants to train predictive models, which perform as well as those trained with the 27D attribute vectors.

## Methods

### Four-body potential and computational mutagenesis

Substantial summaries of these methodologies are discussed below, with finer details available in a previously published manuscript (Masso, 2015). In order to generate robust frequency data for developing the four-body potential, we selected from the PDB a large training set consisting of 1,417 diverse (<30% sequence identity), high resolution (<2.2 Å) X-ray protein structures (Supplemental Information). The structures were coarse-grained at the residue level using the amino acid C-alpha atomic coordinates, which served as vertices for generating a tiling of each protein via the Qhull software implementation of Delaunay tessellation (Barber, Dobkin & Huhdanpaa, 1996), yielding in each case a 3D convex hull containing hundreds of space-filling, non-overlapping, irregular tetrahedra. Each tetrahedron objectively identifies at its vertices a quadruplet of nearest-neighbor interacting residues via their C-alpha atoms by virtue of this computational geometry technique; as further assurance, all edges longer than 12 Å were immediately removed from each protein tessellation prior to further analysis.

Working with a standard protein alphabet, the number of 4-letter subsets that can be enumerated reaches 20^4^ = 160,000 by allowing repetitions within a subset (e.g., AACC) as well as permutations of letters (e.g., AACC, ACAC, CACA, and CCAA are all distinct orderings). In any protein structure tessellation, the four vertices of a tetrahedron may indeed include repeated occurrences of particular residue types; however, since the four vertices are not ordered, permutations of residue quadruplets are excluded, and a single representation (e.g., the four letters in ascending alphabetical order) is enumerated. Thus, in this case the four vertices of a tetrahedron in any protein structure tessellation may represent any one of 8855 distinct types of residue quadruplets. For each such residue quadruplet (*i*, *j*, *k*, *l*), a relative frequency of occurrence *f*_*ijkl*_ was calculated based on the number of times it was observed at the four vertices of a tetrahedron among the 1,417 tessellations. A rate *p*_*ijkl*_ expected by chance for each residue quadruplet was also computed using a multinomial reference distribution, given by

\begin{eqnarray*}{p}_{ijkl}= \frac{4{!}}{\prod _{n=1}^{20} \left( {t}_{n}{!} \right) } \prod _{n=1}^{20}{a}_{n}^{{t}_{n}}, \text{where}\sum _{n=1}^{20}{a}_{n}=1 \text{and}\sum _{n=1}^{20}{t}_{n}=4. \end{eqnarray*}

In the formula above, *a*_*n*_ represents the proportion of all residues found in the training set of 1,417 protein structures that are of type *n*, and *t*_*n*_ is the number of times that residue type *n* is repeated in the quadruplet (*i*, *j*, *k*, *l*). Applying the inverted Boltzmann principle to the data (Sippl, 1993; Sippl, 1995), an energy of interaction score *s*_*ijkl*_ =  − log(*f*_*ijkl*_∕*p*_*ijkl*_) was computed for each residue quadruplet type, and the collection of 8855 scores defines the four-body potential (Supplemental Information).

**Figure 2:**
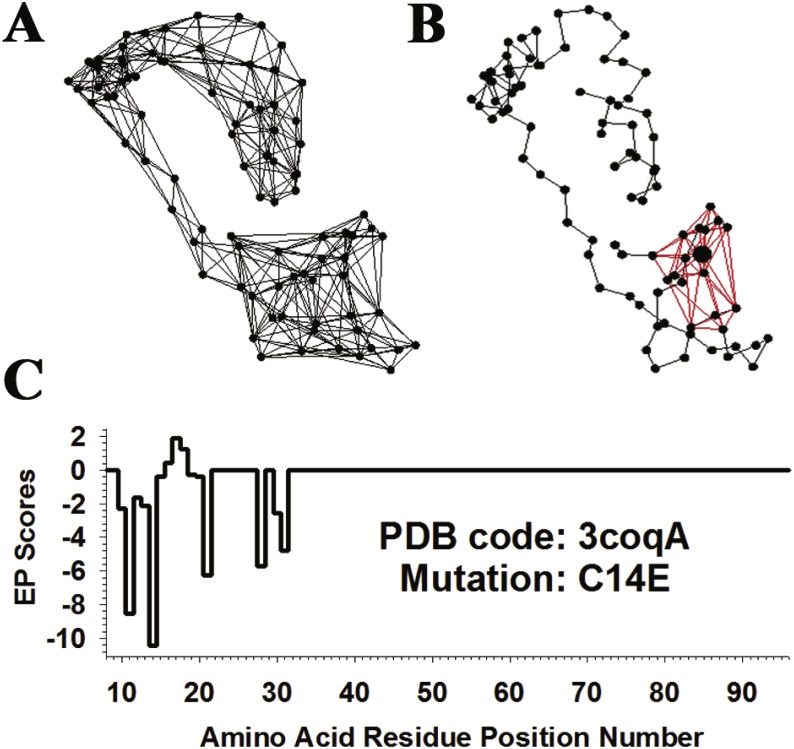
Visualization of the *in silico* mutagenesis methodology. (A) Delaunay tessellation of monomeric Gal4 from Fig. 1C, modified by the removal of tetrahedral edges longer than 12 Å. (B) Twenty-four tetrahedra from the modified tessellation that all share as a vertex the C-alpha coordinate of residue C14, which is enlarged relative to the others. Collectively, there are 14 additional C-alpha vertices that form these tetrahedra, and they represent Gal4 residues forming the tessellation-based local structural neighborhood of C14. (C) Residual profile for the Gal4 variant C14E. The 15 residue positions with nonzero EP scores correspond precisely to the mutated position 14 and its 14 neighbors, and their C-alphas form the 15 total vertices of the tetrahedra shown in (B). Attributes related to mutated position 14 and only its six closest neighbors, as determined by the lengths of tetrahedral edges in (B), are included among the 27 input attributes for the C14E Gal4 variant feature vector.

The *total potential* of any protein with known structure, including Gal4, can be computed using the four-body potential as follows: first, tessellate the C-alpha coordinates of the structure and remove edges longer than 12 Å (Fig. 2A); next, assign a score to each tetrahedron in the tessellation according to the interaction energy score of the residue quadruplet at its four vertices as tabulated in the four-body potential; and finally, add up the scores of all the tetrahedra in the tessellation. Each C-alpha coordinate in a tessellation is typically shared as a vertex by a number of tetrahedra in its immediate surroundings, and a *residue environment score (RES)* can be computed for each residue position in the protein by adding up the scores of all tetrahedra that share its C-alpha coordinate as a vertex; furthermore, a vector of RES scores for all the residue positions in a protein forms a *3D-1D potential profile* (Bowie, Luthy & Eisenberg, 1991).

To analyze single residue substitutions in a protein such as Gal4, we began with the same tessellation as that of the native protein structure and proceeded along similar lines as above, by first replacing the residue associated with the C-alpha coordinate for the position of interest, hence altering the scores of all tetrahedra that share the C-alpha point as a vertex (Fig. 2B). For the mutant protein, RES values were recomputed at all positions in the protein chain, leading to a new 3D-1D potential profile; moreover, the difference between these profiles for the mutant and native proteins is a vector referred to as a *residual profile* for the mutant (Fig. 2C). The scalar components of the residual profile are termed *environmental perturbation (EP) scores*, calculated for each position as the difference between RES values in the mutant and native proteins. For single chain protein structure tessellations in particular, the nonzero EP score at the mutated position in a residual profile is identical to the global *residual score* defined in the Introduction (Masso & Vaisman II, 2007). The only other nonzero EP scores that appear in a residual profile vector in the case of a single chain protein occur at all positions with which the mutated position shares a tessellation edge, quantifying all local effects of the mutation (Fig. 2). For the general case of multimeric protein structure tessellations, the residual score of a mutant is not equivalent to the EP score at any position and must be obtained by taking the difference in total potentials between the mutant and native protein; in a homodimer such as Gal4, for example, a mutation would simultaneously alter the same residue position in both chains. Lastly, for each position in Gal4 a *comprehensive mutational profile (CMP) score* was computed as an average of the residual scores for all 19 residue substitutions at that position, with CMP collectively referring to the vector of CMP scores for all positions.

### Gal4 variant feature vectors, machine learning, and model performance

As previously reported in greater detail (Masso, 2015; Masso & Vaisman II, 2008; Masso & Vaisman II, 2014), the tessellation-based computational mutagenesis approach yields data that allow every single residue protein variant to be uniquely characterized as a feature vector containing 27 input attributes. The method focuses on the mutated residue position and its six closest neighbors in the structure, identified by the lengths of edges between their respective C-alphas in the protein tessellation. We used this technique as a way to represent the Gal4 variants. The use of six nearest neighbors strikes a balance between competing interests: as the required number of neighbors for the residue positions increases, their environments are more accurately modeled; however, the number of positions that are simultaneously excluded for having fewer than the minimum number of neighbors also increases, and feature vectors cannot be generated for any mutations at those positions. For each Gal4 variant, these 27 structural components included:

• position number of the residue substitution;
• the identities of the native and replacement residues;
• EP score at the mutated position;
• the difference in primary sequence numbers between that of the mutated position and those of all six closest neighbors;
• the identities of the residues and the EP scores at the six neighbors;
• the mean volume and mean tetrahedrality for all tetrahedra in the tessellation of Gal4 that share the C-alpha coordinate of the mutated residue as a vertex;
• tessellation-based determination of the mutated position location in the protein (surface, undersurface, or buried) as well as the number of edge contacts it has with surface residue positions;
• the secondary structure at the mutated position.

A final (28th) output attribute at the end of each feature vector consisted of the Gal4 variant function. As the homodimeric structure of Gal4 is subsequently analyzed in the Discussion, the complete residual profiles of the Gal4 variants (EP scores at all positions in each chain) are used exclusively as an alternative set of input attributes for the Gal4 variant feature vectors (Masso & Vaisman II, 2007).

Four classification algorithms—random forest (RF), support vector machine (SVM), decision tree (DT), and neural network (NN), and two regression algorithms—reduced error pruning tree (REPTree) and support vector regression (SVR), were implemented with the Weka (version 3.6) software package (Frank et al., 2004; Witten & Frank, 2000) to train and evaluate predictive models for the Gal4 variants with known functional levels. Parameter selections for each algorithm implementation are provided in the Results. For the regression algorithms, the log_2_ (effect size) functional values for the Gal4 variants were used as the output attributes in their feature vectors (Kitzman et al., 2015). For the classification algorithms, binary functional categories were used owing to the following observations. Based on the three categories of Gal4 variant activity mentioned in the Introduction, we noted a statistically insignificant difference between Gal4 variants that are superior to or equivalent to wild-type (see beginning of Results). This information, combined with data presented by Kitzman et al. (2015) in Table S3, led us to implement a log_2_(effect size) value of −2.0 as an appropriate cutoff between Gal4 variants whose activities are unaffected (≥−2.0) versus negatively affected (< − 2.0) for use as a pair of categorical output attributes in classification algorithms; moreover, the data set consists of 453 unaffected and 631 affected mutants. Gal4 variant activity class predictions made by each of the classification algorithms are based on output probabilities generated for their membership in each of the two functional categories (Witten & Frank, 2000); hence, an additional “combined classifier” was implemented by averaging these probabilities.

Evaluations of model performance were based on cross-validation (CV) results, leave-one-out (LOOCV) as well as tenfold (10-fold CV), and both were implemented in the study. With 10-fold CV, the Gal4 variants (represented as feature vectors to the machine learning algorithms) are randomly stratified to ten disjoint subsets roughly equal in size. With each of 10 iterations, a different subset is held-out for testing (10% of the data) while a model is trained using the combined Gal4 variants from the remaining nine subsets (90% of the data), and the trained model is then used to predict the (known) activities of the Gal4 variants in the test set. Under LOOCV, each Gal4 variant initially forms its own subset (a singleton), then the procedure follows in a manner analogous to that of 10-fold CV. Referring to the two categories of Gal4 variants as negatively affected (P) or unaffected (N) activities, respectively, classifier predictions were evaluated by calculating sensitivity = TP / (TP + FN), specificity = TN / (TN + FP), and PPV = positive predictive value (i.e., precision) = TP / (TP + FP). Performance measures that are robust to differences in category sizes were also computed, including the balanced accuracy rate BAR = 0.5 × [Sensitivity + Specificity], Matthew’s correlation coefficient (MCC), and the area (AUC) under the receiver operating characteristic (ROC) curve. With the regression models, the output attribute for each Gal4 variant feature vector is an activity value as opposed to a category, and they generate numerical predictions. In these instances, in addition to reporting the overall correlation (*r*) between the actual and predicted activity values for the Gal4 variants, all values are subsequently converted to their respective activity categories (unaffected versus negatively affected) based on the threshold described in the previous paragraph, for the purpose of evaluating the performance measures associated with classification.

## Results

Observations presented in this section are based on the analyses of data obtained from the structural tessellation of a single chain of the Gal4 protein (PDB accession code 3coq, chain A), followed by a similar investigation based on the tessellation of a biologically functional Gal4 homodimeric structure (3coq, chains A and B).

### Gal4 structure-function relationships

**Figure 3:**
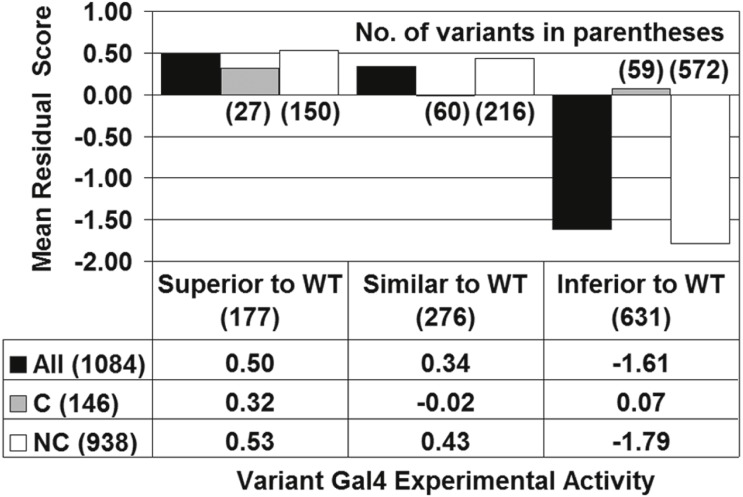
Gal4 structure-function correlation. “All” refers to the full set of 1,084 Gal4 variants, and C/NC are the subsets of these variants that represent conservative/non-conservative amino acid substitutions. The data in the table below the figure are means of the residual scores for the associated subsets of the mutants. All parenthetical whole numbers located either on the graph or in the table row/column headers are counts of the total number of mutants in that subset.

Residual scores were calculated for the 1,084 Gal4 variants with experimentally determined function, and these scores were averaged over all variants in each of the three activity categories described in the Introduction (Fig. 3, black bars). A clear trend emerged, whereby as Gal4 variant function diminished by category, structural effects became increasingly detrimental (lower mean residual scores). The difference in mean residual scores for Gal4 mutants belonging to the Superior/Inferior pair as well as the Similar/Inferior pair of activity categories were each found to be statistically significant (*t*-tests: *p* < 0.0001 for each pair of categories), but this was not the case for the Superior/Similar pair (*p* = 0.14). As discussed in the Methods, this observation supports the use of two functional categories of Gal4 mutants with classification algorithms (Similar and Superior combined versus Inferior). Within each of the three functional categories in Fig. 3, Gal4 mutants were further segregated based on whether the replacement amino acid was a conservative (C) or non-conservative (NC) substitution relative to the native residue, and mean residual scores were computed for each of these subgroups. With the 20 amino acids clustered according to physicochemical similarities as ((A, S, T, G, P), (D, E, N, Q), (R, K, H), (F, Y, W), (V, L, I, M), and (C)), conservative residue substitutions are selected from within the same cluster while inter-cluster replacements are non-conservative (Dayhoff, Schwartz & Orcut, 1978). As reflected by Fig. 3, the overall structure-function relationship is fundamentally driven by non-conservative Gal4 variants, while conservative substitutions minimally impact Gal4 structure on average (mean residual scores are closer to zero for Gal4 variants in each of the C subgroups). In particular, the difference between the mean residual scores for the 27 Superior and 60 Similar conservative (C) substitutions in Fig. 3 is not statistically significant (*p* = 0.12), despite the fact that the mean residual scores of the Gal4 variants in those categories are 0.32 and −0.02, respectively, owing to the relatively small number of mutants.

In support of these results, a contingency table was also generated to analyze the distribution of the residual scores for all 1,084 experimental Gal4 mutants, based on the three functional categories (Superior, Similar, and Inferior) as well as three residual score categories (< − 0.5, [ − 0.5, 0.5], and >0.5). A chi-square test applied to the resulting 3 × 3 table (Supplemental Information; *χ*^2^ = 40, with 4 degrees of freedom) leads to rejection of the null hypothesis that no association exists between activity level and residual scores (*p* < 0.0001).

### Gal4 residue position classifications with *in silico* data

**Figure 4:**
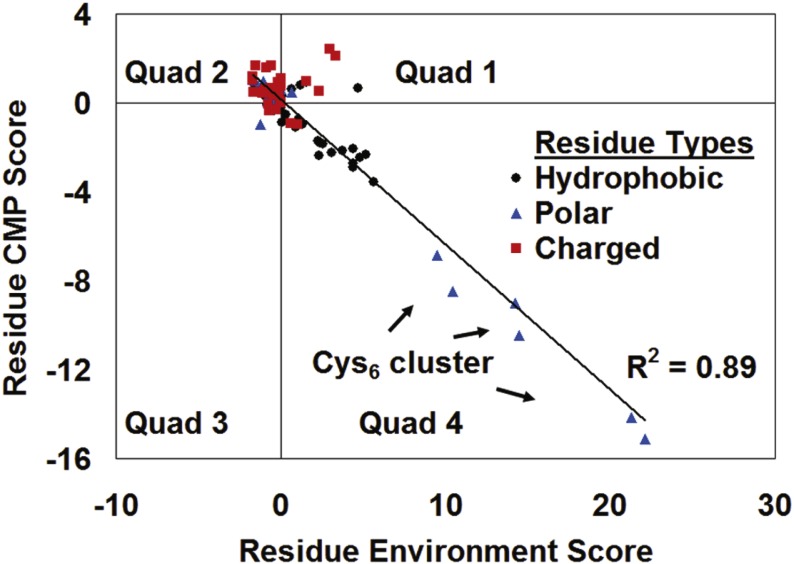
Gal4 CMP-potential profile correlation plot. Note how the amino acids in the protein tend to cluster by their polarity (apolar tend toward Quad 4; charged tend toward Quad 2; and polar cluster about the origin), with the exception of the Cys_6_ binuclear cluster. Hydrophobic residues, especially in the protein core, typically have positive RES scores owing to the network of favorable interactions they form with one another. Substituting these residues, especially with polar or charged amino acids or with residues of significantly different size, leads to unfavorable interactions with the neighbors, and therefore the CMP scores at these positions are negative. Charged residues on the protein surface often interact with other macromolecules or the solvent, and their interactions with other residues in the protein are modestly unfavorable. This explains their negative RES scores and positive CMP scores. Lastly, polar residues are generally ambivalent about their environment and tend to have RES and CMP scores close to zero.

For each residue position in Gal4, the CMP score represents the mean of the residual scores for all 19 possible amino acid substitutions, hence averaging the structural effects of having introducing all possible residues other than the native at that position. The RES score, on the other hand, quantifies the compatibility of the native residue within its structural environment in Gal4. A plot of the CMP scores against the RES scores for the 89 residue positions in a single chain of the Gal4 structure (Fig. 4) revealed a strong inverse correlation (*R*^2^ = 0.89). Furthermore, when the residual scores of non-conservative (NC) and conservative (C) residue substitutions at each position were averaged separately to generate separate NC-CMP and C-CMP scores, the data revealed that NC substitutions (*R*^2^ = 0.88) were primarily responsible for the overall correlation in Fig. 4, while C substitutions (*R*^2^ = 0.02) provided no meaningful contribution. In support of the correlation evident in Fig. 4, a 4 × 3 contingency table was generated (Table 1) to analyze the distribution of all Gal4 residues by quadrant locations (Quads 1–4) as well as by residue polarities (apolar, charged, and polar). A chi-square test applied to the data in Table 1 (*χ*^2^ = 24.8, with 6 degrees of freedom) led to rejection of the null hypothesis that no association exists between polarity and location (*p* < 0.0001).

**Table 1:** Distribution of all Gal4 residues in structure 3coqA.

	Residue types			
Graph quads	Apolar	Charged	Polar	Total
Q1	5	6	2	13
Q2	5	20	15	40
Q3	4	3	1	8
Q4	19	3	6	28
Total	33	32	24	89

**Figure 5:**
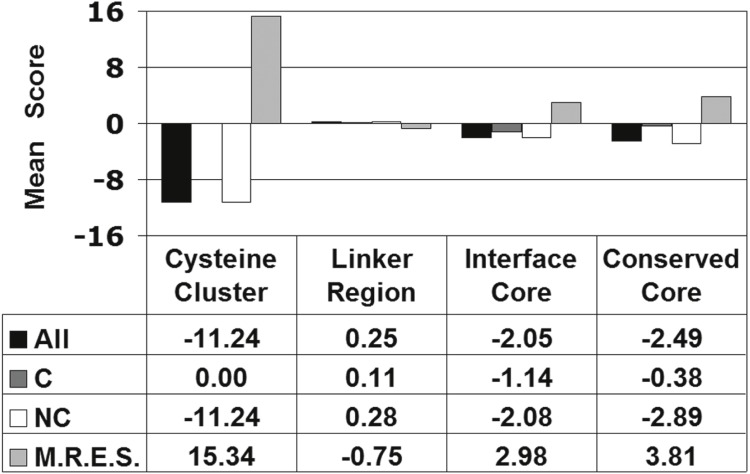
Characteristics of Gal4 structural/functional residue groups. “All” refers to the mean of the residual scores for all Gal4 variants at all positions belonging to each structural/functional group. Whereas the mean of the residual scores for all 19 variants at a single position yields a CMP score for that position, here this concept is generalized by taking the mean of the residual scores for 19 × *N* variants, where *N* is the number of positions in the group. C/NC are the subsets of all variants in each group that represent conservative/non-conservative amino acid substitutions. M.R.E.S. refers to the mean of the residue environment scores for all residue positions in each group. The inverse relationship between the mean of the residual scores for all Gal4 variants in a group (All) and the mean of the residue environment scores for all positions in the group (MRES) stems from the strong inverse correlation observed between CMP and RES in Fig. 4.

To illustrate how the *in silico* data distinguish between groups of Gal4 residue positions based on structural or functional considerations, we analyzed an annotated set of 30 positions that were categorized by Hong et al. as follows: binuclear cluster cysteines (C11, C14, C21, C28, C31, and C38), linker region (S41, P42, K43, T44, K45, R46, S47, P48, and L49), dimer interface core (R60, R63, L64, L67, F68, L70, I71, F72, L77, I80, L81, M83, I89, L92, and L93), and a subset of the dimer interface core that is highly conserved among fungal Gal4 homologs (L67, I71, I80, L81, and L93) (Hong et al., 2008). Table 2 reveals the distribution of the residues belonging to each category based on their Cartesian coordinate quadrant locations in Fig. 4. Using Fisher’s exact test on this 4 × 4 contingency table led to rejection of the null hypothesis that no association exists between residue categories and quadrant locations (*p* < 0.0001). For each category of Gal4 residue positions, Fig. 5 displays the calculated mean of their residue environment scores (M.R.E.S.), as well as the mean of the residual scores associated with the collective set of 19 single residue substitutions taken over all positions in the category (All). As before, these category-wide Gal4 variants were segregated as conservative or non-conservative substitutions, and mean residual scores were obtained for both subgroups (C/NC). Substantial differences exist in mean scores between distinct groups of annotated residues; in particular, the mean scores for the subset of dimer interface positions that are highly conserved among fungal Gal4 homologs are more extreme than the mean scores for all dimer interface residues combined.

**Table 2:** Distribution of annotated Gal4 residues.

	Residue types				
Graph quads	Cysteine cluster	Linker region	Interface core	Conserved core	Total
Q1	0	0	0	0	0
Q2	0	8	0	0	8
Q3	0	0	0	0	0
Q4	6	1	15	5	27
Total	6	9	15	5	35

### Predictive models of Gal4 variant function

Default parameters in Weka were applied here to all machine learning algorithms, with the following exceptions: RF (use 100 trees), SVM (build logistic models and use radial basis function (RBF) kernel), NN (implement one hidden layer), and SVR (use RBF kernel). The algorithms were implemented using the data sets of Gal4 variants represented as feature vectors consisting of 27 input attributes and a single categorical (classification algorithms) or numerical (regression algorithms) output attribute. Tessellation of the Gal4 structure revealed that residue T44 has only five neighbors (edge-lengths <12 Å between the C-alphas of each neighboring residue and T44), fewer than the six neighbors required to generate a complete set of 27 input attributes for each of the 16 Gal4 variants (activities: 13 unaffected, three detrimentally affected) defined by residue substitutions at position 44. In particular, since the missing 6th neighbor would have contributed information for three of the 27 input attributes (residue identity and EP score at the sixth neighbor, and its primary sequence distance from the mutated position 44), feature vectors cannot be generated for these variants. Hence, these 16 variants were excluded, and the data sets each contained a total of 1,068 Gal4 variants.

**Figure 6:**
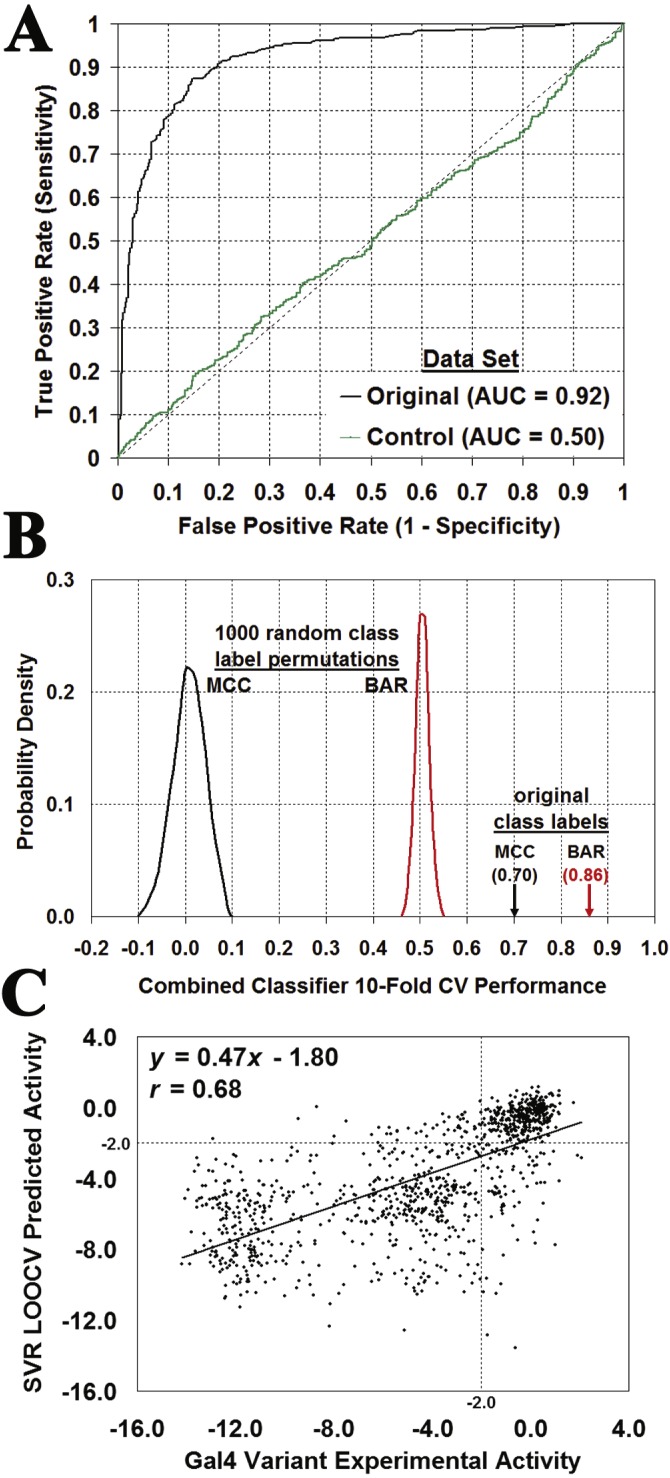
Evaluating the significance of Gal4 prediction performance. (A) Leave-one-out cross-validation (LOOCV) ROC curves obtained with the combined classifier by using the original data set as well as a control set generated by a single random shuffling of the activity class output attributes among the 1068 Gal4 variants in the data set. (B) Distribution of LOOCV combined classifier prediction performance over 1,000 random activity class label permutations, compared with results using the original data set (BAR, balanced accuracy rate; MCC, Matthew’s correlation coefficient). (C) Scatter plot comparing SVR LOOCV predictions obtained for the Gal4 variant activity values versus their experimentally measured values yields a correlation of *r* = 0.68.

Performance results of leave-one-out cross-validation (LOOCV) testing are shown in Table 3. All four classification methods performed equally well, highlighting the robustness of the Gal4 variant data set and lack of bias toward any one machine learning approach. The combined classifier led to improved predictions by averaging prediction probabilities of activity class membership obtained with all four methods. Performance measures for the combined classifier suggest that it successfully embodies the best predictive qualities of the individual models. The following experiment highlighted the significance of these performance results: LOOCV testing was performed using the combined classifier with a control Gal4 variant data set generated by randomly shuffling the 1,068 activity class output attribute labels (440 unaffected/628 affected) among the feature vectors for the Gal4 variants in the original data set. The performance results (AUC = 0.50, BAR = 0.48, and MCC = − 0.06) were indicative of a model that performs no better than random guessing, as highlighted by a graphical comparison of the ROC curves obtained using the combined classifier on both the original and control Gal4 variant data sets (Fig. 6A).

**Table 3:** Prediction performance on 1,068 Gal4 variants. Feature vector input attributes based on *in silico* mutagenesis using the tessellation of one Gal4 monomer (Chain A).

Method	Se	Sp	PPV	BAR	MCC	AUC
LOOCV classification:						
RF	0.87	0.81	0.87	0.84	0.68	0.91
SVM	0.89	0.75	0.84	0.82	0.65	0.88
DT	0.88	0.79	0.86	0.84	0.68	0.86
NN	0.90	0.82	0.88	0.86	0.73	0.88
Combined classifier	0.88	0.83	0.88	0.85	0.71	0.92
LOOCV regression:						
REPTree (*r* = 0.65)	0.91	0.74	0.83	0.83	0.67	—
SVR (*r* = 0.68)	0.92	0.81	0.87	0.87	0.74	—
Predictions made by existing methods:						
SNAP	0.78	0.59	0.73	0.69	0.38	0.69
SIFT	0.93	0.60	0.77	0.76	0.57	0.80

We subsequently employed a more systematic approach for assessing the statistical significance of the combined classifier predictions. In the first step, ten runs of tenfold cross-validation (10-fold CV) testing with the original Gal4 variant data set yielded mean performance values of BAR = 0.86, and MCC = 0.70. Next, 1,000 distinct control data sets were generated by successively shuffling the output attribute class labels among the Gal4 variants in the original data set. The average of 10-fold CV testing over the 1000 control data sets led to performance values of BAR = 0.50 ± 0.01 and MCC = 0.00 ± 0.04. These performance data are summarized in Fig. 6B, suggesting that the *p*-value for predictive power of the combined classifier model is less than 0.001. Furthermore, similar 10-fold CV statistical significance results using the original and 1000 shuffled control data sets were verified using each of the four individual classifiers: RF (original BAR = 0.84, MCC = 0.68; shuffled BAR = 0.50 ± 0.02, MCC = 0.00 ± 0.03); SVM (original BAR = 0.81, MCC = 0.64; shuffled BAR = 0.50 ± 0.02, MCC = 0.00 ± 0.04); DT (original BAR = 0.84, MCC = 0.68; shuffled BAR = 0.50 ± 0.02, MCC = 0.00 ± 0.04); NN (original BAR = 0.86, MCC = 0.71; shuffled BAR = 0.50 ± 0.01, MCC = 0.00 ± 0.03). Next, regression models also performed well under LOOCV testing (Table 3), with performance measures rivaling those of the classifiers. A graphical depiction of the correlation (*r* = 0.68) between actual Gal4 variant activity values and those predicted with the SVR model is presented in Fig. 6C. Table 3 also shows that predictions generated for these 1068 Gal4 variants by the related methods SNAP (Bromberg & Rost, 2007) and SIFT (Sim et al., 2012) are less robust with respect to both sensitivity and specificity, leading to lower performance results over nearly all reported measures. Lastly, none of the predictions obtained using five methods of Pires, et al. discussed in the Introduction correlated well with the experimental Gal4 functional data (0.11 ≤ *r* ≤ 0.26), which they suggested was a reflection of a range of different effects of the mutations on Gal4, including decreased stability and disruptions to both homodimeric interactions as well as interactions with DNA. By linearly combining the predictions from the five models using a regression model tree, they achieved a correlation of *r* = 0.69. Random forest predictions using the five models in combination displayed 81% overall accuracy in Gal4 variant classification with AUC = 0.86.

**Figure 7:**
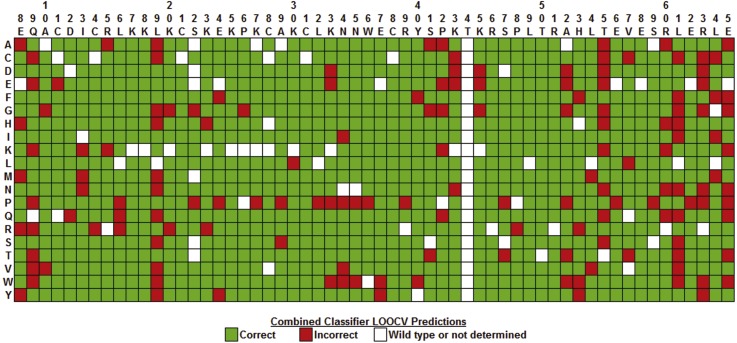
Combined classifier model LOOCV prediction array. The columns identify the wild type residues at positions 8–65 in the Gal4 protein, the rows represent each of 20 types of residue substitutions, and each cell corresponds to the Gal4 variant defined by substituting the native residue at the position in the top column header with the replacement residue given in the left row header. Green and red cells represent Gal4 variants that were predicted correctly and incorrectly, respectively, by the combined classifier model. Cells for which the native residue (column header) and replacement residue (row header) are identical do not represent variants, and these are colored white. Additionally, a few variants for which functional data were not determined were not included in the data set for prediction, and these cells are also colored white. Lastly, predictions were not generated for mutations at position 44, which has fewer than six nearest neighbors because the variants could not be modeled with our computational mutagenesis technique, and these cells are similarly colored white. Prediction errors are noticeable in the dimerization domain (positions 50–65), at positions 8, 9, and 19 within the DNA-binding domain, and for variants defined by the use of proline (P) as a substitution.

An array identifying all of the LOOCV predictions made by the combined classifier for the Gal4 variants is shown in Fig. 7. Note that no predictions were made for single residue substitutions at T44 because this position had fewer than 6 neighbors in the tessellation of the Gal4 structure, precluding the construction of feature vectors for these Gal4 variants and their inclusion in the combined classifier data set. Substitutions with proline create backbone kinks and generated the largest number of incorrect predictions (19/58) scattered throughout the DNA-binding (residues 8–40), linker (residues 41–49), and dimerization (residues 50–65) domains. Furthermore, incorrect predictions were biased toward the dimerization domain relative to the other two domains, which is indicative of a weakness associated with obtaining computational mutagenesis data from the tessellation of an isolated monomer of the Gal4 protein structure. Close interactions between dimer interface residues from both chains were not taken into account here; however, an *in silico* mutagenesis and subsequent analyses based on the tessellation of the complete homodimer are presented below.

### Predictive models incorporating homodimeric Gal4 structure

**Figure 8:**
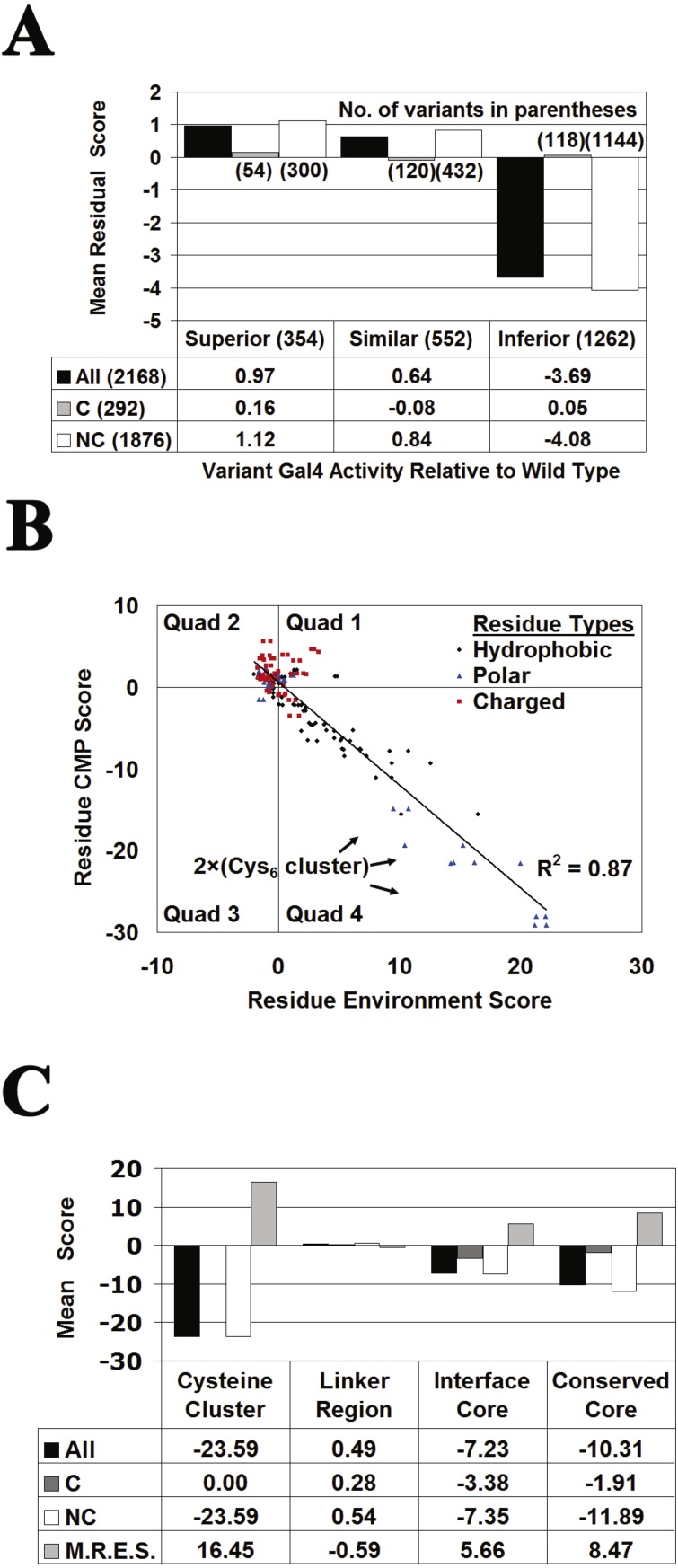
Gal4 structure-function relationships. The structural and computational mutagenesis data used here are based on tessellation of the complete homodimeric structure of Gal4. The plots represent (A) the Gal4 structure-function correlation, (B) the Gal4 CMP-potential profile correlation, and (C) characteristics of Gal4 structural/functional residue groups.

**Figure 9:**
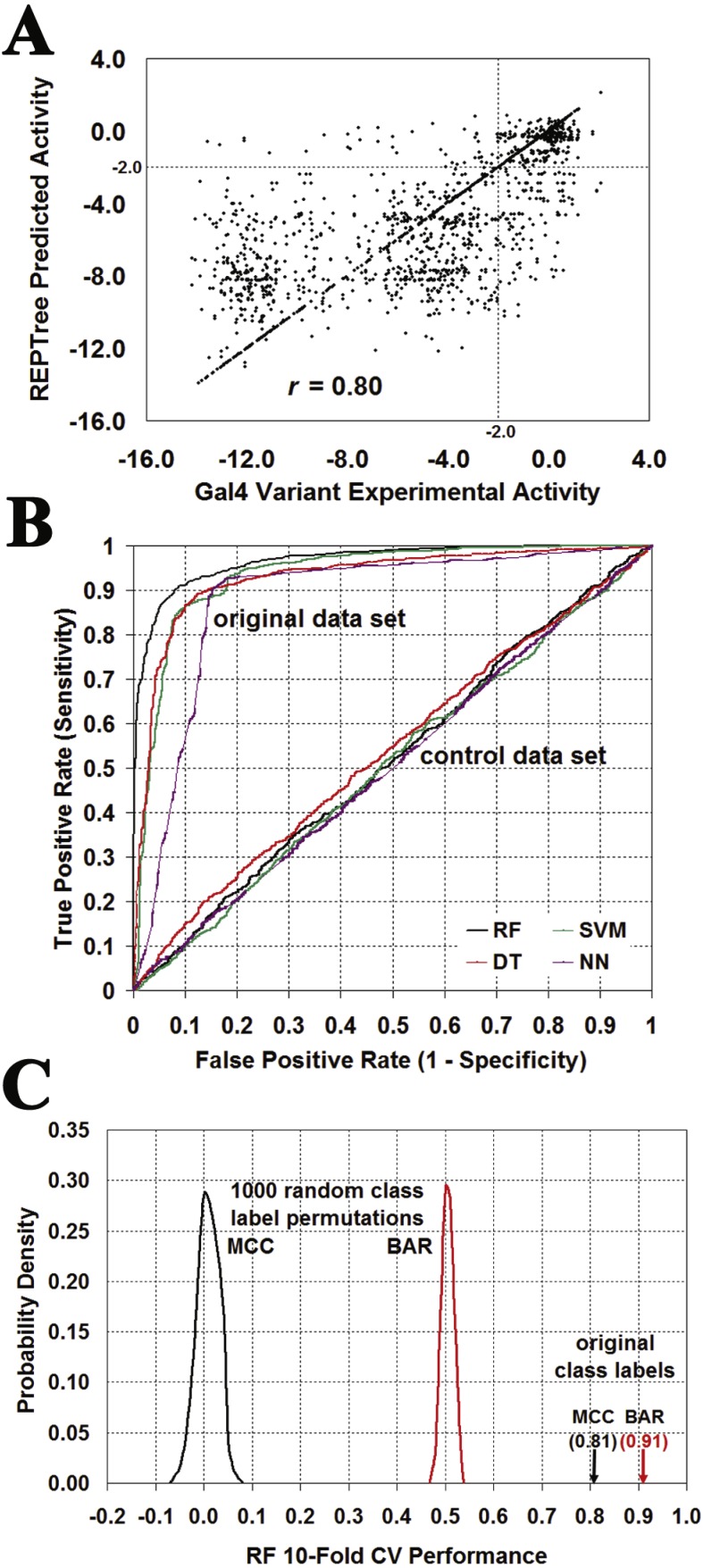
Evaluating the significance of Gal4 prediction performance. Computational mutagenesis that uses the tessellated Gal4 homodimer generates two distinct feature vectors for each Gal4 variant, representing the specific residue substitution at the same position in both monomers. (A) Scatter plot comparing REPTree 10-fold CV predictions obtained for the Gal4 variant activity values versus their experimentally measured values yields a correlation of *r* = 0.80. (B) 10-fold ROC curves obtained with the four classifiers by using the original data set as well as a control set generated by a single random shuffling of the activity class output attributes among the 2136 Gal4 variants in the data set. (C) Distribution of 10-fold CV RF prediction performance over 1,000 random activity class label permutations, compared with results using the original data set (BAR, balanced accuracy rate; MCC, Matthew’s correlation coefficient).

Given the potential for limitations on prediction performance using an isolated monomer of Gal4, especially at dimer interface positions, a parallel study was undertaken with the structural tessellation of the complete homodimeric structure of Gal4 and the subsequent computational mutagenesis. With twice as many residues now in the structure (positions in chain B renumbered to follow those of chain A), and with each residue substitution in Gal4 appearing simultaneously at two positions in the dimer, we proceeded to generate data that included: RES scores for all positions, residual scores and residual profile vectors for all Gal4 variants, and CMP scores for each position. Structure-function relationships and correlations previously observed for the Gal4 monomer (chain A) were strengthened or maintained with data generated from tessellation of the dimeric Gal4 structure (Fig. 8). As before, feature vectors were generated for the Gal4 variants consisting of 27 input attributes and one (numerical or categorical) output attribute; however, since each Gal4 variant now corresponds to a residue substitution at two positions (one in each monomer), two feature vectors were generated, each having a distinct set of input attributes and both sharing the same output attribute. Performance results using both classification and regression data sets of 2,136 feature vectors for Gal4 variants based on 10-fold CV testing are shown in Table 4. Weka default parameters were used for all machine learning algorithms, with the following exceptions: RF (use 100 trees), SVM (*C* = 10.0, build logistic models and use radial basis function (RBF) kernel), DT (average of 10 bagged (bootstrap aggregated) iterations), NN (implement one hidden layer), and SVR (*C* = 10.0, use RBF kernel). Table 4 reflects significant prediction improvements over previous results (Table 3), with the REPTree model performing especially well (Fig. 9A).

**Table 4:** Prediction performance on 2136 Gal4 variants. Feature vector input attributes based on *in silico* mutagenesis using tessellation of the Gal4 dimer.

Method	Se	Sp	PPV	BAR	MCC	AUC
10-fold CV classification:						
RF	0.91	0.90	0.93	0.91	0.81	0.97
SVM	0.91	0.82	0.88	0.87	0.74	0.94
DT	0.89	0.87	0.91	0.88	0.76	0.93
NN	0.91	0.83	0.89	0.87	0.75	0.88
10-fold CV regression:						
REPTree (*r* = 0.80)	0.96	0.86	0.91	0.91	0.83	–
SVR (*r* = 0.72)	0.92	0.84	0.89	0.88	0.77	–

Two control data sets were generated by randomly shuffling the 2136 activity class output attribute labels (classification) or the actual activity values (regression) among the feature vectors for the Gal4 variants in the original data sets. Applying 10-fold CV with these controls performed no better than random guessing, as observed in Fig. 9B for ROC curves obtained using the classification algorithms, and supported with these additional 10-fold CV performance results for all algorithms: RF (BAR = 0.51, MCC = 0.02); DT (BAR = 0.52, MCC = 0.04); SVM (BAR = 0.50, MCC = 0.00); NN (BAR = 0.50, MCC = 0.01); REPTree (*r* =  − 0.02, BAR = 0.50, MCC = 0.03); and SVR (*r* =  − 0.06, BAR = 0.49, MCC = − 0.03). For a more systematic approach to evaluating statistical significance, 1,000 classification and 1000 regression control data sets were generated by random shuffling of the Gal4 variant activity categories and values in the original data sets, respectively, and 10-fold CV testing results employing the original Gal4 variant data sets (Table 4) were compared to those obtained with the control data sets (Fig. 9C displays outcomes with the RF algorithm): RF, SVM, and DT (BAR = 0.50 ± 0.01, MCC = 0.00 ± 0.03); NN (BAR = 0.50 ± 0.01, MCC = 0.00 ± 0.02); REPTree (*r* =  − 0.01 ± 0.03); SVR (*r*= 0.00 ± 0.03). The results in every case suggest that the *p*-value for predictive power of each original model is less than 0.001.

**Figure 10:**
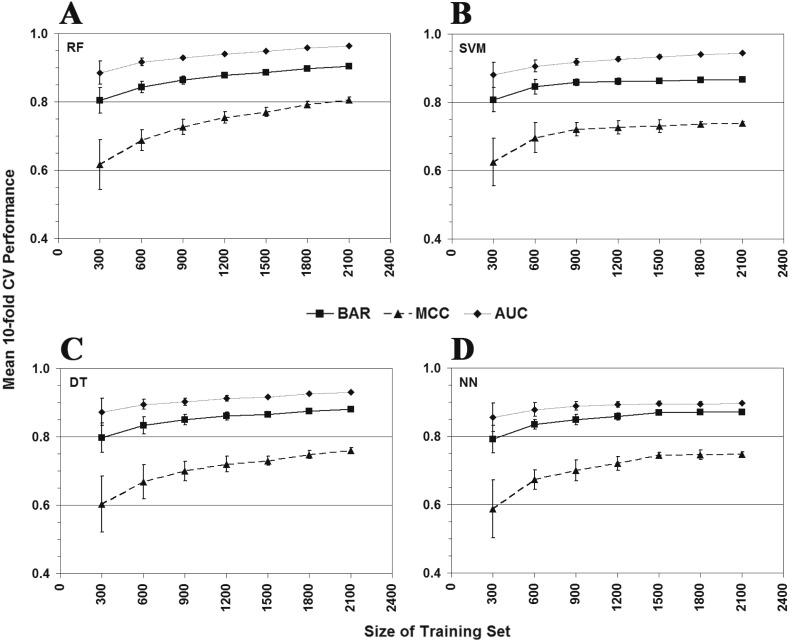
Classifier learning curves. Learning curves made using the (A) random forest, (B) support vector machine, (C) decision tree, and (D) neural network classification algorithms. For each classifier, the plots reveal the degree to which performance is enhanced as the number of Gal4 variants in the training set is increased. Each point represents the average over ten runs of 10-fold CV, and the error bars indicate the standard deviation.

Learning curves (Fig. 10) were subsequently created to visualize the impact of Gal4 variant data set size on model performance. For each classification algorithm, 10-fold CV was applied to ten stratified random samples of 300 Gal4 variants each, with each sample selected from the full set of 2136 Gal4 variants, and mean BAR, MCC, and AUC values and standard deviations were calculated over the ten sets. Subsequent iterations doubled the number of variants selected for each of the ten sets over their previous steps, with the final iteration employing 2,100 Gal4 variants with each sample. The plots reveal that as few as 300 Gal4 residue replacements, well-distributed at positions throughout the structure, are capable of achieving good predictive performance; furthermore, the plots become relatively flat for data sets containing more than 1,200–1,500 Gal4 variants, indicating that a data set smaller than all 2,136 Gal4 variants may be sufficient for training accurate predictive models.

## Discussion

As mentioned at the start of the last section making use of the Gal4 homodimeric structure tessellation, each Gal4 variant in this scenario is represented in the data set by two separate feature vectors. The two vectors have distinctive sets of 27 input attributes corresponding to one particular mutated position located within each of the two separate Gal4 monomers, and both feature vectors having the same activity (category or value) output attribute. These 2,136 Gal4 variant feature vectors in the data set were then separated into two equal-sized subsets according to which Gal4 monomer contained the mutated position representing the feature vector. The two subsets of feature vectors enabled the evaluation of 10-fold CV prediction performance for each isolated chain (A and B) following dimeric tessellation (Table 5). The algorithms all performed well on both subsets, though the prediction results in Table 5 were not as impressive as those obtained with the dimeric data set (Table 4); on the other hand, the 10-fold CV results using both subsets were equivalent to prior LOOCV prediction results based on Gal4 variant feature vectors obtained from tessellation of the isolated A chain (Table 3). To assess the degree of similarity between the two subsets of feature vectors, RF classification and SVR regression models were trained using one subset, and the models were subsequently used for predicting the activity (class or value) output attributes of the Gal4 variants in the other (test) subset based on the 27 input attributes in their respective feature vectors (Table 6). Though the RF models performed well at correctly classifying the Gal4 variants, the SVR models were relatively weaker with respect to specificity (proportion of all unaffected Gal4 variants that were correctly predicted) after converting the predicted activity numerical values to classes based on the −2.0 threshold; hence, differences exist between pairs of feature vectors for the same Gal4 variant occurring at the same position in separate chains, which reflects the sensitivity of protein structure tessellations to the most subtle shifts in C-alpha coordinates.

**Table 5:** Chain-specific Gal4 variant prediction performance. Feature vector input attributes based on *in silico* mutagenesis using tessellation of the Gal4 dimer. The full data set was split into two subsets of 1,068 Gal4 variants each by monomeric chain.

Method	Se	Sp	PPV	BAR	MCC	AUC
A chain, 10-fold CV classification:						
RF	0.87	0.82	0.87	0.84	0.69	0.91
SVM	0.89	0.82	0.88	0.86	0.72	0.89
DT	0.87	0.82	0.88	0.84	0.69	0.90
NN	0.90	0.82	0.88	0.86	0.72	0.87
A chain, 10-fold CV regression:						
REPTree (*r* = 0.64)	0.96	0.59	0.77	0.77	0.61	–
SVR (*r* = 0.66)	0.91	0.83	0.88	0.87	0.74	–
B chain, 10-fold CV classification:						
RF	0.88	0.81	0.87	0.85	0.70	0.91
SVM	0.89	0.83	0.88	0.86	0.72	0.89
DT	0.89	0.83	0.88	0.86	0.72	0.90
NN	0.90	0.82	0.88	0.86	0.72	0.87
B chain, 10-fold CV regression:						
REPTree (*r* = 0.63)	0.93	0.62	0.78	0.77	0.59	–
SVR (*r* = 0.66)	0.90	0.81	0.87	0.86	0.72	–

**Table 6:** Gal4 monomers predict one another. Feature vector input attributes based on *in silico* mutagenesis using tessellation of the Gal4 dimer. The full data set was split into two subsets of 1,068 Gal4 variants each by monomeric chain. Variants from one chain were predicted using a model trained with the variants from the other chain.

Method	Se	Sp	PPV	BAR	MCC	AUC
A chain—training/B chain—testing:						
RF	0.96	0.71	0.82	0.83	0.71	0.96
SVR (*r* = 0.60)	0.95	0.54	0.75	0.75	0.56	–
B chain—training/A chain—testing:						
RF	0.96	0.85	0.90	0.90	0.82	0.98
SVR (*r* = 0.66)	0.94	0.56	0.75	0.75	0.56	–

As the algorithm reporting the best performance results (Table 4), the RF 10-fold CV predictions for Gal4 variants were further segregated based on the depth and secondary structure of the mutated residue positions (Table 7), as well as the polarities of the native and replacement amino acid residues of the Gal4 variants (Table 8). Of the 27 input attributes for each Gal4 variant feature vector, the non-EP score attributes assist predictions by characterizing the structural location (e.g., depth) and environment (e.g., secondary structure) of the mutated position, while EP scores at the mutated position and its six closest neighbors assist predictions by characterizing the effects of each type of residue replacement at that position.

**Table 7:** Mean RF 10-fold CV prediction performance based on depth and secondary structure.

	BAR	MCC	%
**Depth**			
Buried	0.90	0.81	65
Undersurface	0.96	0.91	12
Surface	0.86	0.74	23
**Secondary Structure**			
Helix	0.91	0.81	49
Coil	0.91	0.81	51

**Table 8:** Mean RF 10-fold CV prediction performance based on side chain polarities of the native and new amino acids at the mutated position.

New/native	Polar			Apolar			Charged			All		
	BAR	MCC	%	BAR	MCC	%	BAR	MCC	%	BAR	MCC	%
Polar	0.92	0.84	13	0.89	0.75	11	0.92	0.85	6	0.91	0.81	30
Apolar	0.88	0.78	13	0.88	0.75	8	0.93	0.85	6	0.89	0.79	27
Charged	0.92	0.85	20	0.91	0.81	16	0.84	0.68	7	0.90	0.81	43
All	0.91	0.83	46	0.90	0.78	35	0.89	0.79	19	0.90	0.81	100

**Figure 11:**
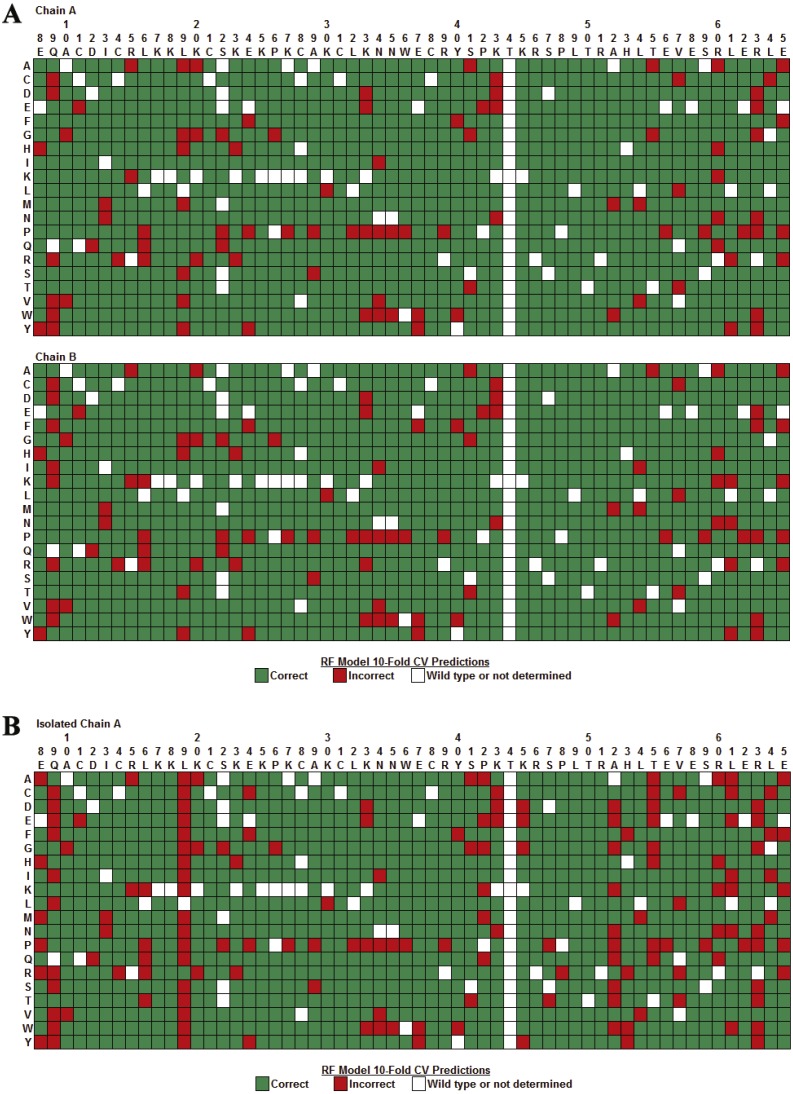
Comparing prediction performance arrays. (A) RF 10-fold CV predictions with a Gal4 dimeric feature vector data set, consisting of 2,136 variants, obtained by computational mutagenesis that uses the tessellated Gal4 homodimer. Each Gal4 variant is represented by two distinct feature vectors in the data set, corresponding to the specific residue substitution at the same position in both monomers, which can form two equally-sized subsets of 1,068 Gal4 variant feature vectors, one for each chain. (B) RF 10-fold CV predictions with the subset of 1,068 Gal4 feature vectors for chain A of the Gal4 dimer. Significant improvements are observed when Gal4 variant predictions in chain A are generated as part of predictions for variants from both chains as opposed to in isolation.

An array identifying 10-fold CV predictions made by the RF classifier (Table 4) for all 2,136 Gal4 variants based on the tessellated dimer is shown in Fig. 11A. Similarly, Fig. 11B shows 10-fold CV predictions made by the RF classifier (Table 5) for the subset of 1068 Gal4 variants corresponding to the isolated A chain subsequent to dimeric tessellation. The array in Fig. 11A reflects significantly fewer errors in the dimerization domain (residues 50–65) of both chains relative to the array for the isolated A chain in Fig. 11B, which more closely resembles the array obtained from LOOCV predictions made by the RF classifier using a data set of Gal4 feature vectors generated by tessellating the monomeric A chain (Fig. 7). For positions 50–65, the isolated A chain (Fig. 11B) generated 61 incorrect predictions, while the A chain as part of a complete dimer (Fig. 11A) led to only 31 incorrect predictions. Significant prediction improvements occurred at residues A52, T55, L61, and L64 as well as modest improvements at H53 and R63. These corrections at interface positions introduced into RF algorithm learning via tessellation of the complete dimeric structure also have a positive influence on predictions at residues E8, Q9, and L19 that interact with the DNA, a molecular structure which cannot be directly modeled with this residue-based approach. Regarding the combined predictions for both chains of Gal4 (Fig. 11A), 103 of the 199 total incorrect predictions were due to substitutions creating substantial structural rearrangements: sharp backbone kinks, P (32 errors); bulky aromatic or long side chains: W (16 errors), R (15 errors), Y (13 errors); very small or no side chains: G (14 errors), A (13 errors). The remaining types of residue substitutions displayed fewer errors. Results presented in Fig. 11 strongly support modeling and analyses based on computational mutagenesis data derived from the tessellation of a biologically functional form of the protein structure under consideration, which in this case is the Gal4 homodimer.

Up to this point, the Gal4 variants used to train all models have been represented as feature vectors consisting of 27 input attributes characterizing the structure (and sequence) of the local environment surrounding the mutated position. Among these attributes are the EP scores for only the mutated position and its six closest neighbors, obtained from the residual profile of the mutant. By selecting only seven EP score components from the residual profile vectors, and supplementing them with 20 additional features, the goal was to employ a diverse and universally applicable set of attributes to fully characterize local environments of mutated positions. As a final exercise based on the dimeric tessellation of Gal4, we considered the complete residual profile vectors as an alternative to the set of 27 input attributes for the Gal4 variant feature vectors. In particular, recall that each Gal4 variant defines the same residue substitution at two C-alpha positions simultaneously in the dimeric tessellation, from which the computational mutagenesis generates a single residual profile vector consisting of EP scores at all 178 positions in the dimer (residues 8–96 for both chains sequentially). The first (last) 89 components of this 178D vector correspond to the EP scores for every position in the A chain (B chain) due to the simultaneous mutation at the same position in both chains; hence, the 178D vector was split to form two 89D residual profile vectors of EP scores representing the same variant in the two chains. These 89D vectors of EP scores were used as alternative input attributes for the feature vectors representing the Gal4 variants, generating a data set of 2,168 Gal4 feature vectors (16 Gal4 variants at position 44 in each chain that previously could not be represented using the approach of 27 input attributes can now be included) for training predictive machine learning models. Three additional input attributes, corresponding to a variant ID (wild-type residue, position number, and replacement residue), were added to each feature vector to create a second data set. The top half of Table 9 summarizes 10-fold CV performance results using these data sets with classification and regression algorithms. Lastly, the 2,168 Gal4 feature vectors in each of these two data sets were split into two equally-sized subsets of 1,084 Gal4 feature vectors by their Gal4 chain membership. Results obtained by using one subset for model training and the other subset for testing (prediction) are shown in the bottom half of Table 9. All performance results in Table 9 are consistent with prior results using the 27 input attributes and indicative of accurate and reliable predictive models.

**Table 9:** Gal4 variant prediction performance with residual profile vector input attributes.

Method	Input attributes	Se	Sp	PPV	BAR	MCC	AUC
10-fold CV classification:							
RF	EP scores	0.93	0.89	0.92	0.91	0.82	0.97
	EP scores + variant ID	0.97	0.95	0.97	0.96	0.92	0.99
SVM	EP scores	0.83	0.69	0.79	0.76	0.53	0.85
	EP scores + variant ID	0.89	0.87	0.90	0.88	0.75	0.93
DT	EP scores	0.91	0.88	0.91	0.89	0.79	0.95
	EP scores + variant ID	0.90	0.88	0.91	0.89	0.78	0.96
NN	EP scores	0.78	0.79	0.84	0.79	0.56	0.82
	EP scores + variant ID	0.81	0.79	0.84	0.80	0.59	0.83
10-fold CV regression:							
REPTree	EP scores (*r* = 0.72)	0.90	0.75	0.84	0.83	0.67	–
	EP scores + variant ID (*r* = 0.80)	0.94	0.78	0.85	0.86	0.74	–
SVR	EP scores (*r* = 0.53)	0.86	0.62	0.76	0.74	0.50	–
	EP scores + variant ID (*r* = 0.72)	0.91	0.77	0.84	0.84	0.69	–
A chain—training/B chain—testing:							
RF	EP scores	0.95	0.91	0.94	0.93	0.86	0.98
	EP scores + variant ID	0.99	0.98	0.99	0.98	0.97	1.00
REPTree	EP scores (*r* = 0.63)	0.78	0.81	0.85	0.79	0.58	–
	EP scores + variant ID (*r* = 0.63)	0.94	0.56	0.75	0.75	0.56	–
B chain—training/A chain–testing:							
RF	EP scores	0.93	0.91	0.93	0.92	0.83	0.98
	EP scores + variant ID	0.98	0.98	0.98	0.98	0.96	1.00
REPTree	EP scores (*r* = 0.65)	0.75	0.83	0.86	0.79	0.57	–
	EP scores + variant ID (*r* = 0.60)	0.94	0.54	0.74	0.74	0.54	–

## Conclusion

In summary, structural tessellation of a Gal4 monomer combined with a computational mutagenesis that relies on a 4-body potential were used to quantify environmental changes in Gal4 upon single residue substitutions. Structure-function relationships in Gal4 were revealed as these data were compared with experimental measurements of activity changes upon mutation. Additionally, these data were used to generate Gal4 variant feature vectors for training predictive models of Gal4 variant activity using a variety of machine learning algorithms, with relatively consistent results. The fact that Gal4 is functional as a homodimer and contains a dimerization domain led to a reassessment, whereby a substantial improvement in results was observed upon taking into consideration the tessellation of the complete dimeric structure of Gal4. In particular, structure-function correlations were strengthened, and significant improvements were observed in model predictions for Gal4 variants at the dimer interface, which also positively influenced predictions within the DNA binding domain. The methodology is widely applicable to the development of predictive models for other proteins with solved structures for which diverse training sets of variants with known function are available.

##  Supplemental Information

10.7717/peerj.4844/supp-1 Supplemental Information 1 Residual scores only for Gal4 variants with activity values based on monomeric A chain tessellation

10.7717/peerj.4844/supp-2 Supplemental Information 2 Residual scores only for Gal4 variants with activity values based on dimeric tessellation

10.7717/peerj.4844/supp-3 Supplemental Information 3 All Gal4 variant residual scores based on dimeric tessellation

10.7717/peerj.4844/supp-4 Supplemental Information 4 Feature vectors for Gal4 variants with known activity, using 27 input attributes, based on monomeric A chain tessellation

10.7717/peerj.4844/supp-5 Supplemental Information 5 Feature vectors for Gal4 variants with known activity, using 27 input attributes, based on dimeric tessellation

10.7717/peerj.4844/supp-6 Supplemental Information 6 Feature vectors for Gal4 variants with known activity, using residual profile vectors as input attributes, based on dimeric tessellation

10.7717/peerj.4844/supp-7 Supplemental Information 7 All Gal4 variant residual scores based on monomeric A chain tessellation

10.7717/peerj.4844/supp-8 Supplemental Information 8 Training set for the four-body potential A list of Protein Data Bank (PDB) 4-character codes for 1417 diverse, high-resolution protein structures that were tessellated in order to generate the four-body potential. For single chain structures, the symbol ’@’ appears after the PDB code; for multimers, a single capital letter representing the chain selected from the structure file appears after the PDB code.

10.7717/peerj.4844/supp-9 Supplemental Information 9 Four-body potential Energy of interaction scores were calculated for all residue quadruplets.

10.7717/peerj.4844/supp-10 Supplemental Information 10 Programs for deriving the four-body potential Two Perl scripts and instructions for reproducing the four-body potential tabulated in the file potential1417cut12.txt.

10.7717/peerj.4844/supp-11 Supplemental Information 11 Contingency table of Gal4 variant counts based on residual scores and functional categories

## Additional Information and Declarations

### Data Availability

The following information was supplied regarding data availability: The raw data and code are provided in the Supplemental Files.
