# Supplementary material for: Modeling transcriptional activation changes to Gal4 variants via structure-based computational mutagenesis
Source: PeerJ. 2018 May 29;6:e4844. doi: 10.7717/peerj.4844 (PMC5983003; doi:10.7717/peerj.4844)
Supplement: Supplemental Information 11 [file peerj-06-4844-s011.doc]

Table 1. Contingency table of Gal4 variant counts based on residual scores and functional categories.

| Residual Score (RS) | log_2_ (effect size) relative to WT | | | Total |
| --- | --- | --- | --- | --- |
|  | Superior | Similar | Inferior |  |
| RS < -0.50 | 26 | 52 | 213 | 291 |
| -0.50 ≤ RS ≤ 0.50 | 74 | 125 | 231 | 430 |
| RS > 0.50 | 77 | 99 | 187 | 363 |
| Total | 177 | 276 | 631 | 1084 |
